# PURA syndrome—a genetic cause of a neurodevelopmental disorder—case report

**DOI:** 10.3389/fped.2025.1607213

**Published:** 2025-07-10

**Authors:** Jacek Kobak, Mateusz Szczupak, Karolina Czerkiewicz, Sergiusz Bielocerkowski, Sabina Krupa-Nurcek

**Affiliations:** ^1^ Department of Otolaryngology, Faculty of Medicine, Medical University of Gdańsk, Gdańsk, Poland; ^2^ Department of Anesthesiology and Intensive Care, Copernicus Hospital, Gdańsk, Poland; ^3^ Student of Department of Surgery, Institute of Medical Sciences, Medical College of Rzeszów University, Gdańsk, Poland; ^4^ Department of Orthopedic and Spinal Surgery, Medical University of Gdańsk, Rzeszów, Poland; ^5^ Department of Surgery, Institute of Medical Sciences, Medical College of Rzeszów University, Rzeszów, Poland

**Keywords:** PURA syndrome, PURA gene, neurodevelopment disorders, genetic disease, genetic defect

## Abstract

**Introduction:**

PURA syndrome is a rare genetic disorder first described in the medical literature in 2014. It is caused by pathogenic variants in the PURA gene, which is located on chromosome 5. The PURA gene is crucial for the production of the pur-α protein, which is expressed in all tissues, including the nervous system, muscles, and blood. The pur-α protein plays a vital role in normal brain development. The estimated incidence of PURA syndrome is 1 in 1,000,000, and as of 2024, approximately 706 cases of the syndrome have been identified worldwide.

**Aim of study:**

The aim of the study was to present a case description of PURA syndrome and the genetic basis of the neurodevelopmental disorder in a 15-year-old girl.

**Case report:**

This manuscript presents the case of a 15-year-old girl of Polish descent diagnosed with PURA syndrome through genetic testing. She was admitted to the Department of Orthopedics and Spine Surgery at the Medical University of Gdansk for surgical treatment of advanced idiopathic scoliosis caused by a postural defect.

**Conclusion:**

PURA syndrome is a rare genetic condition that requires further research and observation. Although it shares many clinical features with other neurological disorders, certain symptoms—such as speech disorders, the ability to follow and execute simple commands, and an excessive acoustic reaction to surprises—should raise suspicion of this condition. These indicators should prompt genetic testing for confirmation and the implementation of appropriate multidisciplinary care for the patient.

## 1 Introduction

PURA syndrome is a neurodevelopmental disorder inherited in an autosomal dominant manner, classified as a rare disease (1). It is listed in the OMIM (Online Mendelian Inheritance in Man) database with the identifier #616158 (2). The PURA gene (Purine-rich element-binding protein A) encodes the Pur-α protein, which has regulatory functions in processes such as DNA repair and replication, as well as mRNA transport and translation (3, 4). This protein plays a crucial role in proper brain development after birth, including the formation of new synaptic connections, maturation of dendrites, and the proliferation of neural cells (5–8). PURA syndrome is caused by a heterozygous pathogenic sequence variant in the PURA gene, which is located on chromosome 5q31 (9). As of October 2024, approximately 706 cases of PURA syndrome have been confirmed worldwide across 60 countries (10). A total of 317 pathogenic variants of the PURA gene have been identified, with the most common variant being p.Phe233del (10). *de novo* mutations are most common (9, 11). PURA syndrome is characterized by moderate to severe developmental delays, including challenges in motor skills and speech. Individuals with this syndrome may experience hypothermia, hypotonia (reduced muscle tone), apnea (breathing interruptions), feeding difficulties, excessive hiccups, uncoordinated eye movements, visual disturbances, dystonia (involuntary muscle contractions), dyskinesia (difficulty with movement) and scoliosis, which occurs in 48% of cases (1, 9, 12–14). The individual displays facial features that may be considered dysmorphic (15). Less commonly, PURA syndrome may be associated with congenital heart defects, endocrine disorders, genitourinary malformations, and skeletal abnormalities (1, 9, 12, 13). A mutation in the PURA gene is linked to an autosomal dominant form of intellectual disability type 31 (OMIM:616158).

The majority of individuals affected by PURA syndrome are nonverbal, and many are unable to move independently (9). Approximately half of these patients experience epilepsy, with seizures typically beginning in infancy or early childhood; however, the age of onset can vary significantly (5). The syndrome is associated with a high incidence of epilepsy. There is an unclear relationship between genotype and phenotype in PURA syndrome, as patients with identical genetic variants can exhibit a wide range of symptoms and severity. Despite significant advances in molecular genetics, the complete clinical characteristics and epidemiological profile of PURA syndrome are still not fully understood (14, 16, 17). It is important to note that no cases from several continents have been documented in the existing scientific literature (14, 16, 17). The aim of the study was to present a case description of PURA syndrome and the genetic basis of the neurodevelopmental disorder in a 15-year-old girl.

**Figure 1:**
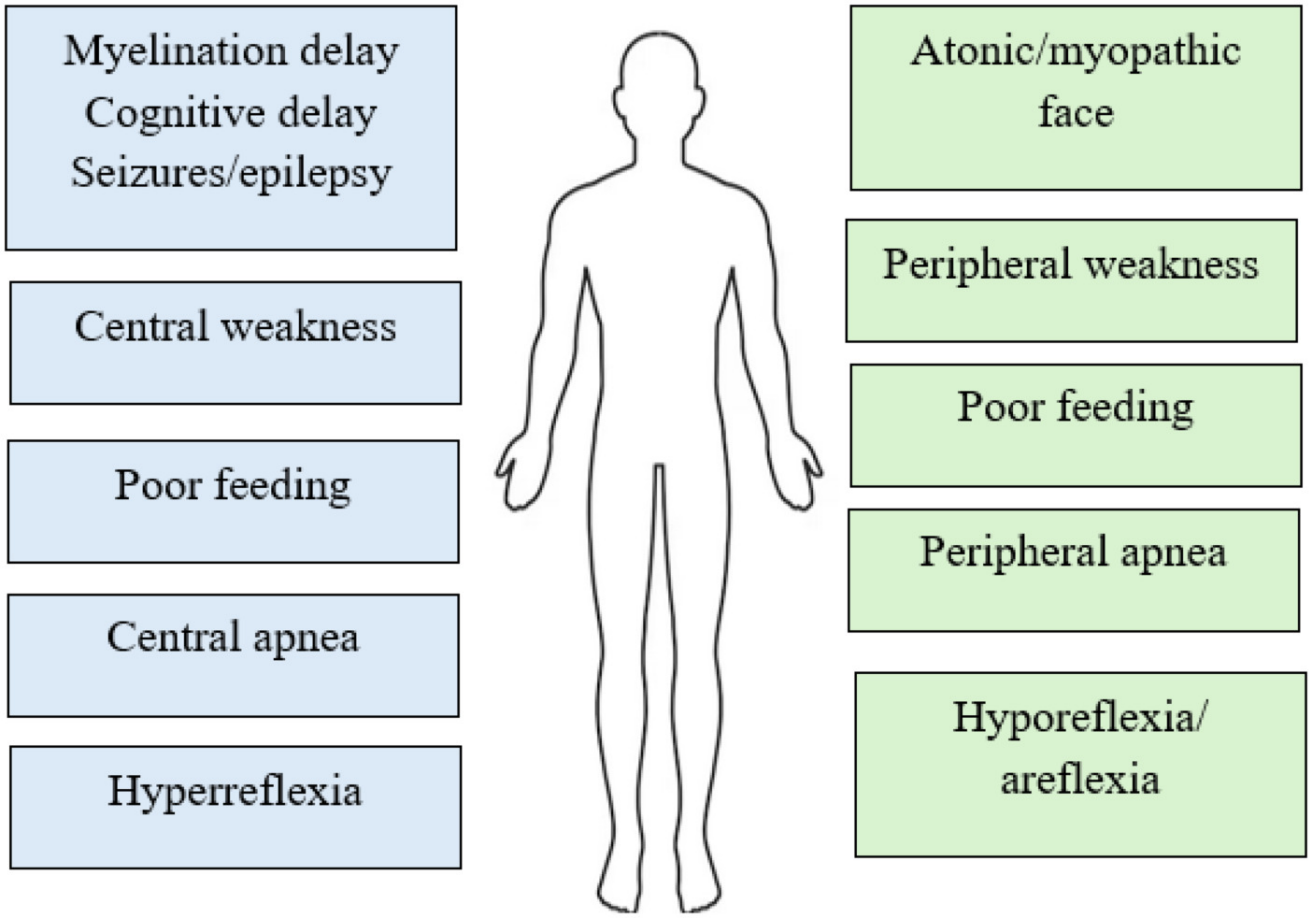
Central vs. peripheral symptoms in PURA (30).

Taniguchi et al. conducted a systematic review to explore the genotype-phenotype correlations in neurodevelopmental disorders associated with PURA syndrome. The authors found that patients with the 5q31.3 deletion syndrome experienced a higher incidence of congenital malformations, respiratory difficulties, and gait issues. In the case of PURA syndrome, variants that cause protein shortening, such as nonsense or frameshift mutations, were linked to increased speech deficits. Interestingly, the location of the PURA variant did not influence the occurrence of congenital defects or neurodevelopmental outcomes (18). Symptoms in PURA are presented on Figure 1.

## 2 PURA protein structure

**Figure 2:**
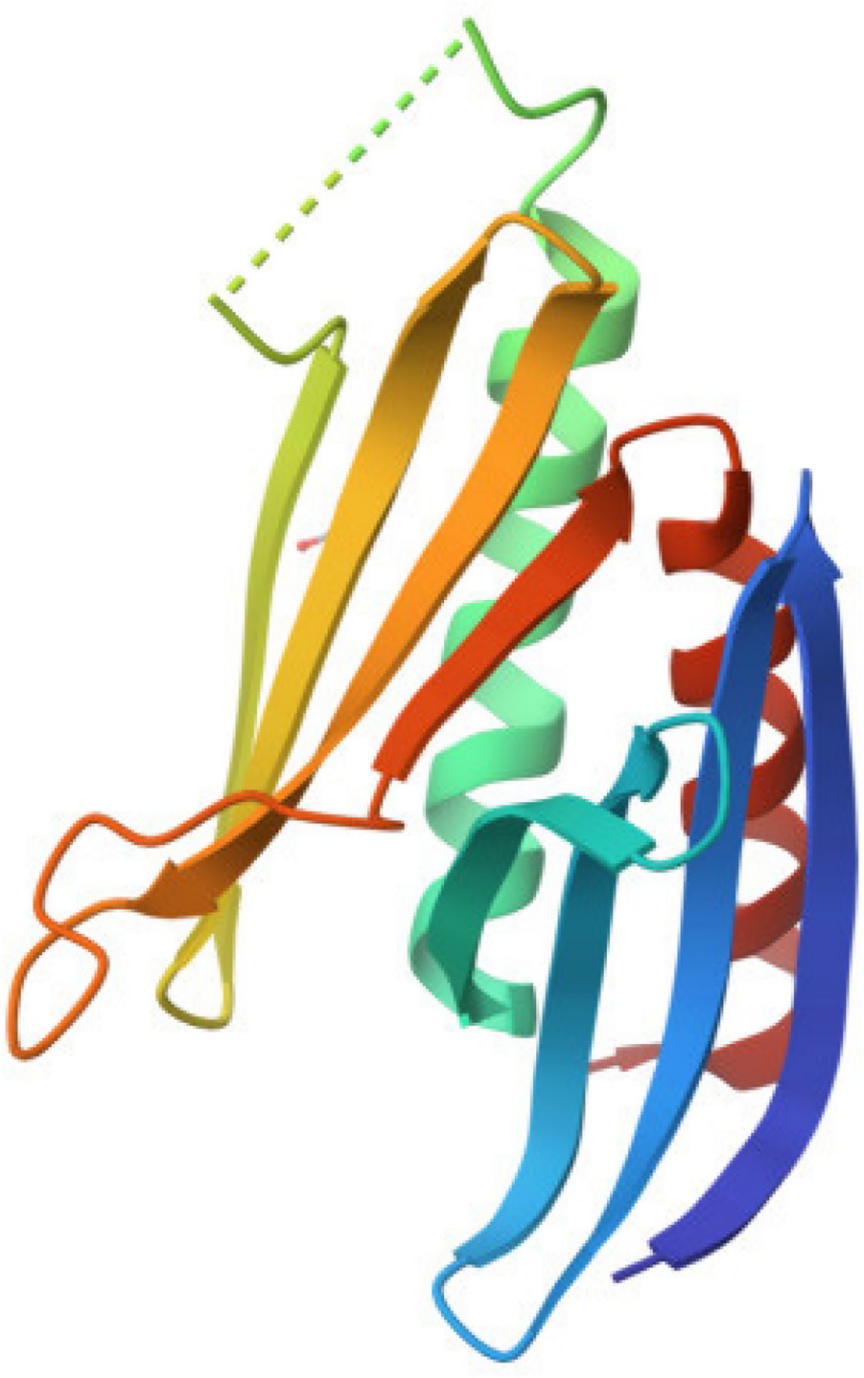
Graphic diagram of the PURA protein structure (44).

PURA protein (purine-rich element binding protein A) is a protein consisting of 322 amino acids with repeated nucleic acid-binding domains (15). It is encoded by the PURA gene located on chromosome 5 (5q31) (4, 19). The PURA protein is primarily located in the cell nucleus and cytoplasm of neurons (4, 6, 20–22). The molecular weight of the PURA protein is 35–37 kDa. There are three domains of this protein, the so-called PUR repeats, namely PUR I, PUR II and PUR III (4). In humans, the PUR domain typically consists of 55–70 amino acids. Each of these domains can bind both DNA and RNA (23). Additionally, PUR they domains participate in dimer formation and interact with other proteins (24) Notably, the structure of the PURA protein lacks classical domains such as the helix-turn-helix (HTH) motif or a zinc finger (Figure 2). One of its key structural features is a high content of *α*-helical motifs, along with a tertiary structure that enables flexible nucleotide binding and involvement in various cellular processes (25). Pur-alpha has been demonstrated to bind to both single- and double-stranded nucleic acids that contain GGN motifs (4). Neuronal DNA/RNA binding protein Pur-alpha is a regulator of transcription and a major factor in mRNA localization (4). In addition to its DNA and RNA binding abilities, Pur-alpha also exhibits dsDNA destabilizing activity in an ATP-independent manner (26). Pur-alpha-mediated binding of nucleic acids involves three central PUR repeats, which are flanked on the N-terminal side by unstructured glycine-rich sequences and on the C-terminal side by regions rich in glutamine and glutamate (27). PUR-alpha interacts with the expanded CGG repeats of FMR1 RNA (28). It is assumed that FMR1 mRNA expression with abnormal trinucleotide repeat expansions is a major cause of neurodegenerative tremor/ataxia syndrome associated with a fragile X chromosome (11).PUR-alpha also interacts with the expanded GGGGCC repeat RNAs arising from hexanucleotide repeat expansions in the first intron of the c9orf72 transcript (29)**.** These interactions of PUR-alpha protein with repeat RNAs lead to sequesteration and loss of PUR-alpha protein function. This loss of function contributes to the pathogenesis of Fragile X-associated tremor/ataxia syndrome (FXTAS) and amyotrophic lateral sclerosis/frontotemporal dementia (ALS/FTD), respectively (4, 22).

The table below provides information on which selected RNA the Pur-alpha protein binds to and its function (Table 1).

**Table 1:** The selected RNA binds to the Pur-alpha protein, and it is important to understand what function this interaction serves.

Type of RNA	Examples	Function of interaction	Source
mRNA	Myelin Basic Protein mRNA, Amyloid Precursor Protein mRNA, Microtubule-Associated Protein 1B mRNA	Transport to dendrites, local translation, regulation of protein synthesis in neurons	(45)
Viral RNA	Trans-Activation Response (TAR) element of Human Immunodeficiency Virus Type 1, Regulatory RNA of JC Virus	Regulation of viral transcription, replication, and RNA stability	(46)
Pathological repeat RNA	Expanded GGGGCC hexanucleotide repeats in Chromosome 9 open reading frame 72 (C9orf72)	Binding to toxic RNA repeats, protection from neurotoxicity associated with ALS/FTD	(46)
Long Non-Coding RNA (lncRNA)	Brain Cytoplasmic RNA 1 (BC1), Brain Cytoplasmic RNA 200 (BC200)	Regulation of mRNA localization and translation in neurons	(45)
Structured regulatory RNA	RNA G-quadruplexes, stem-loop (hairpin) structures	Recognition of RNA secondary structures, control of translation efficiency	(47)
Stress granule-associated RNA	Various mRNAs recruited to stress granules during cellular stress	Formation and maintenance of stress granules, translational arrest under stress conditions	(46)
RNA associated with fragile X mental retardation protein (FMRP)	G-quadruplex-rich neuronal RNAs bound by FMRP	Co-regulation of translation in neuronal projections through RNA–protein complexes	(48)

## 3 Aim of the study

The aim of the study was to describe the physical and neurodevelopmental presentation of a 15 year old with PURA syndrome.

## 4 Material and methods

To write this manuscript, a review of articles was conducted using the PubMed, Google Scholar, and Mendeley search engines. The keywords used were “Pura syndrome,” “intellectual disability,” and “scoliosis.” From the articles found and analyzed, those deemed relevant to the topic of this manuscript and valuable as sources of information were selected. The manuscript cites 54 publications and scientific reports. Additionally, it presents the case of a 15-year-old female patient who required surgical treatment in the Department of Orthopedics and Spine Surgery at the Medical University of Gdańsk due to an advanced postural defect, specifically scoliosis.

## 5 Case presentation

**Figures 3 and 4:**
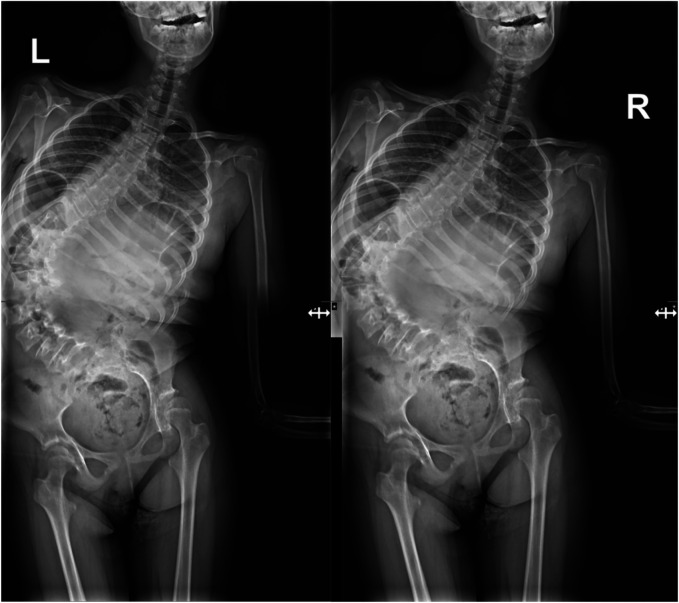
Radiological imaging study showing spinal deformity before surgical treatment.

**Figure 5:**
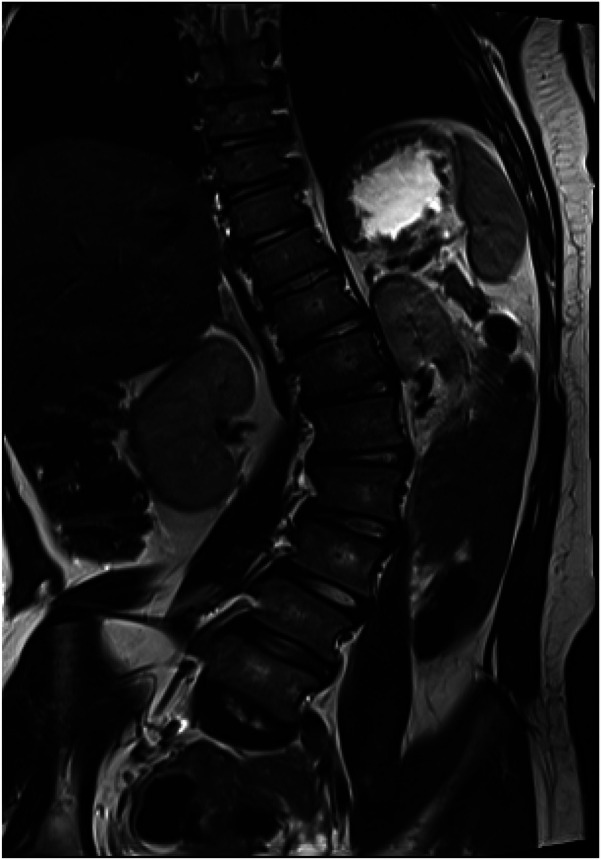
Computer tomography scan before surgical treatment.

A 15-year-old girl was admitted to the Department of Orthopedics and Spine Surgery at the Medical University of Gdansk, where she was diagnosed with PURA syndrome, confirmed by genetic testing. She was admitted due to an advanced postural defect in the form of idiopathic scoliosis (Figures 3–5). This condition significantly impacted her quality of life and caused considerable difficulties in maintaining proper posture and movement.

**Figures 6 and 7:**
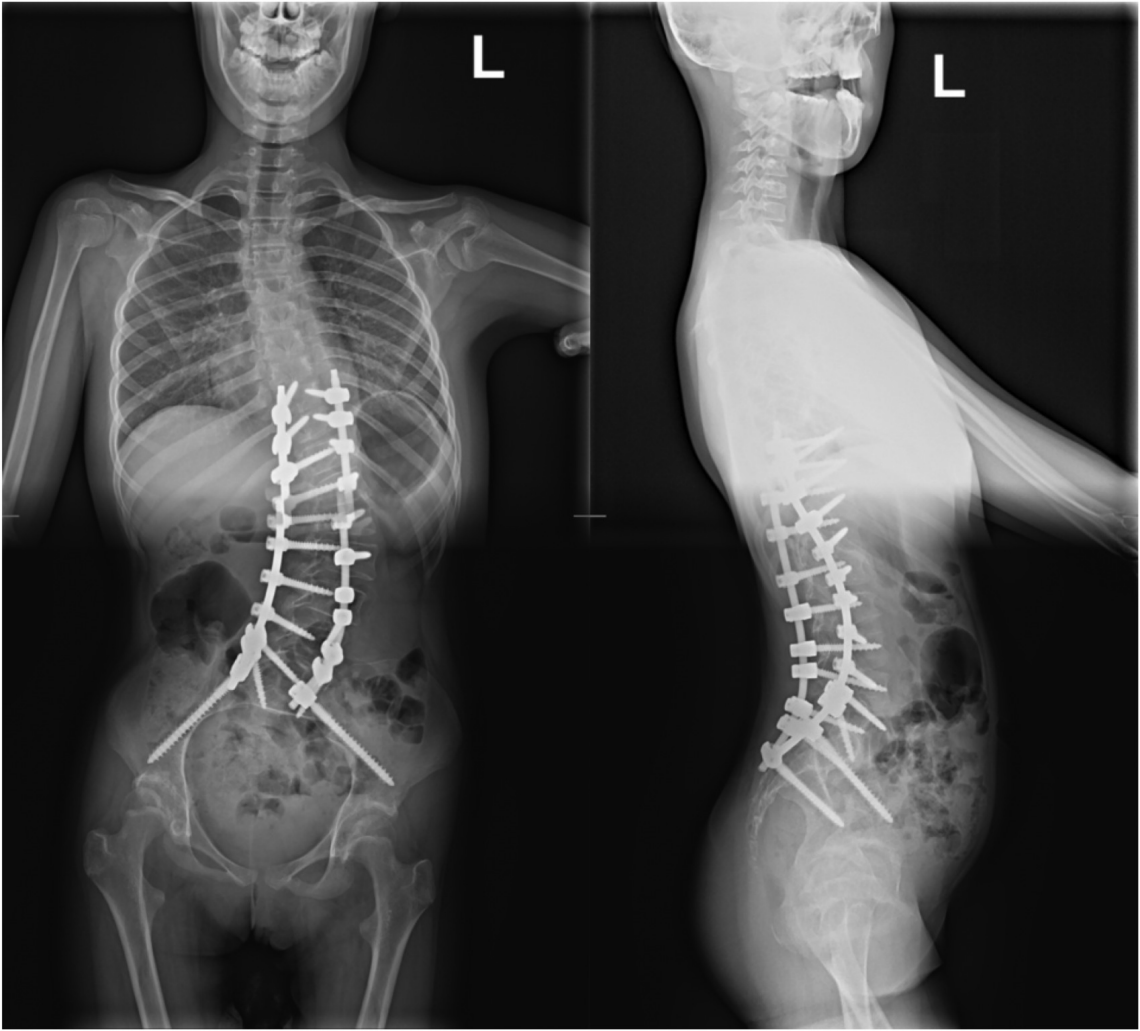
Toposcan after surgical treatment of idiopathic scoliosis.

A girl was born to young, healthy, and unrelated parents, with no history of genetic diseases in the family and no exposure to harmful environmental factors. She was a product of an uncomplicated first pregnancy, delivered naturally at 41 weeks, with a birth weight of 3,800 grams. She scored 8 points on the Apgar scale. In the early childhood period, the child showed symptoms in the form of motor development disorders (difficulty lifting the head in the neonatal period, upper and lower limbs), involuntary movements, unstable gait on a broad base, as well as verbal communication disorders (the child only utters sounds, does not speak single words). Facial dysmorphia, uncoordinated eye movements, and hypotonia of the trunk muscles were also observed. Additionally, feeding problems and symptoms of gastroesophageal reflux were present, including a persistent cough (without other features of an upper respiratory tract infection), regurgitation of gastric contents, and empty belching. Episodes of epileptic seizures were and still are present—for this reason, the girl is treated with valproic acid. Due to delays in psychomotor development and decreased muscle tone from infancy, she was referred to a genetic counseling center for further evaluation. During the diagnostic process, chromosomal abnormalities were examined, revealing a normal karyotype of 46, XX. Conditions such as Prader-Willi syndrome and spinal muscular atrophy were excluded. Molecular studies conducted using next-generation sequencing identified a mutation in one allele of the PURA gene when the girl was 8 years old. Specifically, there was a single nucleotide change (c.470T > G) in exon one, resulting in a missense mutation that alters methionine to arginine (p.Met157Arg). Bioinformatics analysis indicated that this mutation is pathogenic and of *de novo* origin.

In the Orthopedic and Spine Surgery clinic, after properly preparing the patient and obtaining the necessary consents from the girl's legal guardian for the proposed surgical treatment and anesthesia, the procedure of posterior correction and posterolateral stabilization was performed. The surgical procedure and anesthesia were completed without complications. During the recovery period, the child did not experience any neurological deficits and was rehabilitated successfully, resulting in a satisfactory postoperative outcome. The girl was discharged from the clinic on the fifth day after the operation for continued outpatient care. Nine weeks post-operation, the patient attended a follow-up visit at the trauma and orthopedic surgery clinic. An imaging study, a toposcan of the spine, was conducted (see Figures 6, 7). According to the consulting orthopedic surgeon, the results of the operation were satisfactory. The girl continued her rehabilitation program and gradually regained her mobility, which her parents noted significantly improved her quality of life.

## 6 Discussion

Our case illustrates the defining features of PURA syndrome, such as hypotonia, motor and speech delays, epilepsy, and scoliosis, as outlined in the literature (19, 30–34). However, it is important to note that symptoms can vary among individuals. For instance, Liu et al. (14) reported a newborn who experienced feeding difficulties, lethargy, and respiratory failure—symptoms that were not present in our patient. Additionally, while some patients may show signs of multi-organ involvement, this was not evident in our case. PURA syndrome is typically diagnosed in childhood, but the age at which individuals are diagnosed can vary from infancy to adulthood. One study indicated that the average age of diagnosis is 7.4 years, with the youngest patient being just 11 months old and the oldest being 27 years old (14). Conditions that should be considered in the differential diagnosis include Prader-Willi syndrome, myotonic dystrophy, spinal muscular atrophy, and congenital muscular dystrophy (35). Ongoing research suggests that there may be morphological abnormalities associated with the central nervous system (36) including inappropriate acoustic responses to unexpected sounds. For instance, Mroczek et al. (37) described variations in nerve fiber size and rapidly progressive muscular atrophy, which aligns with our patient's presentation of decreased muscle tone and muscular atrophy. Common characteristics of PURA syndrome include abnormal movements and varying severity of epilepsy over time (32). Epilepsy is a frequent occurrence in PURA syndrome, affecting around 50% of patients (33, 34). Our patient has been diagnosed with epilepsy and is currently being treated with valproic acid. The severity and types of seizure activity can differ significantly, highlighting the phenotypic diversity associated with PURA syndrome. Several studies have indicated that apnea is particularly prevalent among newborns with PURA syndrome (11–13) and may serve as a diagnostic indicator. However, apneas were not observed in our case. Skeletal abnormalities, such as scoliosis, are significant features of PURA syndrome (38). In our patient, the worsening scoliosis negatively impacted thoracic organ function, which was evident through shallow breathing and deterioration in gas exchange, as confirmed by acid-base balance analysis. Similar cases have reported gastrointestinal disorders, such as reflux and constipation, likely stemming from impaired motility (15, 39–41). Though diagnosing PURA syndrome can be challenging, the presence of characteristic symptoms should prompt appropriate testing to confirm the diagnosis.

## 7 Conclusion

PURA syndrome is a rare genetic disorder characterized by various systemic and organ-related symptoms. It includes specific features such as speech disorders, difficulty following simple commands, inadequate acoustic responses to unexpected sounds, and feeding difficulties starting from the neonatal period. These symptoms should prompt genetic testing to confirm the diagnosis and allow for appropriate multidisciplinary therapeutic management.

Early and rapid genetic testing in children with developmental disorders is important because it makes it possible to implement early psycho-motor rehabilitation and early detection of additional complications, which in some diseases coexist with a genetic disease. Thanks to genetic testing, we can predict whether the next child also has a risk of developing the disease. In addition, parents can prepare for the presence of additional diseases and faster intervention is possible (18, 38, 42, 43, 49–51).

## Data Availability

The datasets presented in this article are not readily available because of ethical and privacy restrictions. Requests to access the datasets should be directed to the corresponding author.
